# Osteogenic effects of rapamycin on bone marrow mesenchymal stem cells via inducing autophagy

**DOI:** 10.1186/s13018-023-03616-9

**Published:** 2023-02-22

**Authors:** Yifeng Xing, Chaowei Liu, Lin zhou, Yan Li, Dong Wu

**Affiliations:** 1 Fujian Key Laboratory of Oral Diseases & Fujian Provincial Engineering Research Center of Oral Biomaterial & Stomatological Key Lab of Fujian College and University, School and Hospital of Stomatology, Fujian Medical University, Fuzhou, China; 2 Institute of Stomatology & Research Center of Dental and Craniofacial Implants, School and Hospital of Stomatology, Fujian Medical University, Fuzhou, China

**Keywords:** Rapamycin, Osteogenic differentiation, Autophagy, 3-Methyladenine, Bone marrow mesenchymal stem cells

## Abstract

**Background:**

While autophagy is essential for stem cells’ self-renewal and differentiation, its effect on bone marrow mesenchymal stem cells (BMSCs) remains unclear. This study aimed to investigate the interaction between autophagy and osteogenic differentiation using rapamycin (RAPA), a classical autophagy agonist with osteo-regulatory effects.

**Methods:**

Rat BMSC’s autophagy was analyzed after osteoinduction (0, 7, 14, and 21 d) by western blotting, immunofluorescence, and real-time quantitative polymerase chain reaction (RT-qPCR). In addition, we evaluated osteogenic differentiation using alizarin red staining, alkaline phosphatase assays, and RT-qPCR/Western blotting quantification of bone sialoprotein, type 1 collagen, alkaline phosphatase, osteopontin, and Runt-related transcription factor 2 mRNA and protein levels.

**Results:**

The BMSC’s basal autophagy level gradually decreased during osteogenic differentiation with a decrease in BECN1 level and the lipidated (LC3-II) to unlipidated (LC3-I) microtubule-associated protein 1 light chain 3 ratio and an increase in the expression of selective autophagic target p62. In contrast, it increased with increasing RAPA concentration. Furthermore, while 2 nM RAPA promoted BMSC osteogenic differentiation on days 7 and 14, 5 nM RAPA inhibited osteogenesis on days 14 and 21. Inhibition of autophagy by the inhibitor 3-methyladenine could impair RAPA’s osteogenesis-enhancing effect on BMSCs.

**Conclusions:**

The BMSC’s basal autophagy level decreased over time during osteogenic differentiation. However, an appropriate RAPA concentration promoted BMSC osteogenic differentiation via autophagy activation.

## Introduction

Bone defects caused by osteoporosis, inflammation, and trauma are one of the most intractable problems in clinical treatment [1–3]. Therefore, finding effective bone repair methods remains challenging. Therefore, some disciplines such as regenerative medicine and tissue engineering have increasingly relied on cell and tissue cultures to produce and amplify specific cells to replace important differentiation functions lost or altered in different disease states (e.g., bone defects caused by diseases such as osteoporosis) for bone regeneration [4, 5]. BMSCs have been widely applied in the fields of tissue engineering and regenerative medicine. They play crucial roles in bone regeneration therapy due to their multi-directional differentiation potential and ability to differentiate into tissues such as bone, cartilage, and fat under specific conditions [6, 7]. Interestingly, recent findings have demonstrated that autophagy regulation in BMSCs represents a possible molecular mechanism by which to influence their properties, playing a pivotal role in their bone regeneration and therapeutic potential in various biological processes [8, 9].

Autophagy refers to intracellular vesicles that envelop misfolded proteins and dysfunctional organelles and fuse with lysosomes to form autolysosomes, which degrade their contents for nutrient or energy production to maintain their basal metabolism and organelle balance [10]. Accumulating evidence suggests that mesenchymal stem cells’ (MSCs) self-renewal and differentiation processes require strict control of protein turnover and organelle number. Autophagy can rapidly and efficiently degrade substances such as enzymes and transcription factors, providing cells with the anabolic precursors and energy needed to support the morphological, structural, and metabolic reconstruction required for differentiation [11, 12]. Therefore, it is critical for regulating cellular differentiation. The specific role of autophagy and its involvement in BMSCs’ osteogenic differentiation, however, remain unknown.

Previous studies indicated that altered basal autophagy levels may be involved in regulating the adipogenic or osteogenic differentiation process of MSCs. Pantovic and colleagues [13] confirmed the involvement of autophagy in the early osteogenic differentiation of dental pulp-derived MSCs. They observed a transient autophagy induction after differentiation initiation and a subsequent sustained decline in autophagy following differentiation. Another study reported that autophagy activation was also observed in BMSCs at the initial lipogenic differentiation stage, peaking on day 3, followed by a rapid decline on day 7 [14]. In addition, autophagy’s pivotal role in osteogenic differentiation is that undifferentiated MSCs contain many autophagosomes, which are significantly reduced in later differentiation stages [15]. However, the specific changes in BMSC basal autophagy levels at more detailed differentiation stages, including undifferentiated, early, middle, and late periods, have not been investigated to date to the best of our knowledge. Moreover, whether osteogenic differentiation can be affected by pharmacologically altering the autophagy level in this process remains unknown.

Rapamycin (RAPA), a classical mammalian target of RAPA (mTOR) inhibitor, induces autophagy by binding to mTOR, activating the mTOR signaling pathway [16]. Studies have shown that RAPA increases autophagy and affects osteogenic differentiation in MSCs [17–19], but the specific biological mechanisms by which it acts remain unclear. The US Food and Drug Administration, moreover, has approved RAPA for treating humans (e.g., for post-transplant immunosuppression in humans), enabling its conversion for specific orthopedic applications. Therefore, how RAPA might be applied in orthopedics needs to be explored.

Given autophagy’s role in MSC osteogenic differentiation and RAPA’s specific osteomodulatory effects potentially related to autophagic regulation, we investigated basal autophagy levels in BMSCs during the osteogenic differentiation process. In addition, RAPA’s effects on osteogenic differentiation and autophagy were examined. We show that RAPA has potential applications in treating bone defects.

## Methods and materials

### Cell culture

BMSCs were extracted from the bone marrow of male Sprague–Dawley rats (4 weeks old; Shanghai Laboratory Animal Center, Shanghai, China; License No. SCXK20170012) sacrificed by cervical dislocation. Briefly, the bone marrow cavities of the rats’ femur and tibia were rinsed with α-modified Eagle’s medium (α-MEM; Gibco, USA) containing 10% fetal bovine serum (FBS; Cyagen Oricell, China) and 1% penicillin and streptomycin (Beyotime, China) in a sterile environment. Then, the bone marrow cells were cultured in a constant-temperature incubator (5% CO_**2**_, 37 °C). The culture medium was changed every 2–3 d. The cells at passage three (P3) were used for subsequent experiments.

### BMSC identification

First, 1 × 10^6^ cells were digested with trypsin and mixed with 500 µL of PBS containing 5 µL antibodies against cluster of differentiation 34 (CD34), 45 (CD45), 90 (CD90), and 105 (CD105; Abcam, USA). After 40 min of incubation at 4 °C, cells were washed twice and resuspended in 100 µL PBS. Finally, the marker protein was analyzed using the BD Accuri C6 flow cytometer (BD Biosciences, USA).

### Osteogenic differentiation analysis

#### Alkaline phosphatase (ALP) activity

BMSCs were seeded into 12-well plates at a density of 5 × 10^4^ cells/well using osteoblast-inducing conditional medium (α-MEM with 10% FBS, 1% penicillin–streptomycin, 10 mM β-glycerophosphate, 10 nM dexamethasone, and 50 µg/mL ascorbic acid) as the culture medium. Cells were cultured in osteoblast-inducing medium that contained RAPA (Abmole, USA) at concentrations of 0, 2, or 5 nM. All cultures were maintained for a period of 21 days, and 3-MA (Abmole, USA; 5 mM) was added in different experiments. After 7-, 14-, and 21-d of culturing, cells were stained with the BCIP/NBT ALP Staining Kit (Beyotime, China). In addition, Alkaline phosphatase activity was quantitatively analyzed by an ALP Assay Kit (Beyotime, China).

#### Alizarin red staining (ARS)

Cell culture conditions were as described above. RAPA (0, 2, and 5 nM) and 3-MA (5 mM) were added in different experiments. After 14- and 21-d of culturing, cells were stained with 1% alizarin red dye (Solarbio, China) for 15 min and observed using a stereomicroscope (Carl Zeiss, Germany). Then, the calcific nodules were dissolved with 10% cetylpyridinium chloride (Boc Sciences, USA) and quantitated at 560 nm using a microplate reader (Molecular Devices, USA).

### Cell viability

BMSCs at densities of 2 × 10^3^ cells/well were seeded into 96-well plates and cultured for 1, 3, and 5 d. The culture medium contained different RAPA concentrations (0, 2, 5, and 10 nM). RAPA’s effects on cell proliferation were assessed using Cell Counting Kit (CCK)-8 solution (Dojingdo, Japan). Briefly, the CCK-8 solution was added to each well. The optical density was measured at 450 nm using a spectrophotometer.

### RT-qPCR analysis

Relative cytokine gene expression was quantified using RT-qPCR. Cells were cultured in osteoblast-inducing medium that contained RAPA (Abmole, USA) at concentrations of 0, 2, or 5 nM. All cultures were maintained for a period of 21 days, and 3-MA (5 mM) was added in different experiments. Total RNA was isolated from BMSCs using TRIZOL reagent (TaKaRa, Japan). The concentration of total RNA was determined by UV spectroscopy. Then, the genomic DNA was excluded using the PrimeScript™ RT Reagent kit with gDNA Eraser (Takara, Japan) at 42 °C for 2 min. According to the manufacturer’s protocols, RNA was reverse-transcribed into cDNA using the Prime Script RT kit (Takara, Japan). The target genes’ expression was analyzed using the SYBR Green Kit (TaKaRa, Japan) with a Roche 480 Light Cycler (Roche, Germany). The cycling conditions were as follows: incubation at 95 °C for 30 s, 40 cycles of 95 °C for 5 s, and 60 °C for 30 s. The primers used are listed in Table 1.

**Table 1:** Primer sequences for RT-qPCR

Gene	Forward primer sequence (5'-3’)	Reverse primer sequence (5'-3’)
*Gapdh*	ACGGCAAGTTCAACGGCACAG	GAAGACGCCAGTAGACTCCACGAC
*Alpl*	CGTTTTCACGTTTGGTGGCT	ACCGTCCACCACCTTGTAAC
*Ibsp*	ACAACACTGCGTATGAAACCTATGAC	AGTAATAATCCTGACCCTCGTAGCC
*Runx2*	CAGATTACAGATCCCAGGCAGAC	AGGTGGCAGTGTCATCATCTGAA
*Spp1*	GAGCAGTCCAAGGAGTATAAGC	AACTCGTGGCTCTGATGTTC
*Col1a1*	CGAGTATGGAAGCGAAGGTT	CTTGAGGTTGCCAGTCTGTT
*Lc3*	GCGAGTTGGTCAAGATCATCC	CGTCTTCATCCTTCTCCTGTTC
*Becn1*	AATCTAAGGAGTTGCCGTTGT	GCCTCCAGTGTCTTCAATCTT
*Atg5*	GAAGGCACACCCCTGAAATG	CCTCAACTGCATCCTTGCAC

### Western blotting analysis

Relative cytokine protein expression was detected using western blotting. Cells were cultured in osteoblast-inducing medium that contained RAPA (Abmole, USA) at concentrations of 0, 2, or 5 nM. All cultures were maintained for a period of 21 days, and 3-MA (5 mM) was added in different experiments. Proteins were extracted from BMSCs with RIPA (Beyotime, China) on ice. Proteins were loaded and separated by SDS-PAGE. The primary antibodies targeted ALP (1:500; Abcam, USA), Runt-related transcription factor 2 (RUNX2; 1:500; HUABIO, China), bone sialoprotein (BSP; 1:500; Bioss, China), microtubule-associated protein 1 light chain 3 (LC3; 1:500; Proteintech, China), Beclin 1 (BECN1; 1:500; Wanleibio, China), sequestosome 1 (SQSTM1/p62; 1:500; Bioss, China), and GAPDH (1:5000; Abcam, USA).

### Immunofluorescence staining

After RAPA treatment (0, 2, or 5 nM) for 0, 7, 14, and 21 d, BMSCs were fixed in 4% paraformaldehyde, permeated with 0.2% Triton X-100 (pH 7.4) for 10 min, and blocked with 5% bovine serum albumin. The cells were subsequently incubated with an anti-LC3 antibody (Proteintech, China) overnight at 4 °C and treated with a fluorescent secondary antibody (Abcam, USA) for 1.5 h. Next, the nucleus was stained using DAPI ( Beyotime, China). Finally, images were acquired with an Axio Observer A1 inverted fluorescence microscope (Carl Zeiss, Germany).

### Statistical analysis

Each experiment in this study was repeated in triplicate. All the data are expressed as mean ± standard deviation (SD). Data were assessed using Student’s t-test or one-way analysis of variance with the SPSS 19.0 software (SPSS Inc., Chicago, IL, USA). All results with *p* < 0.05 were considered statistically significant.

## Results

### BMSC identification

**Fig. 1:**
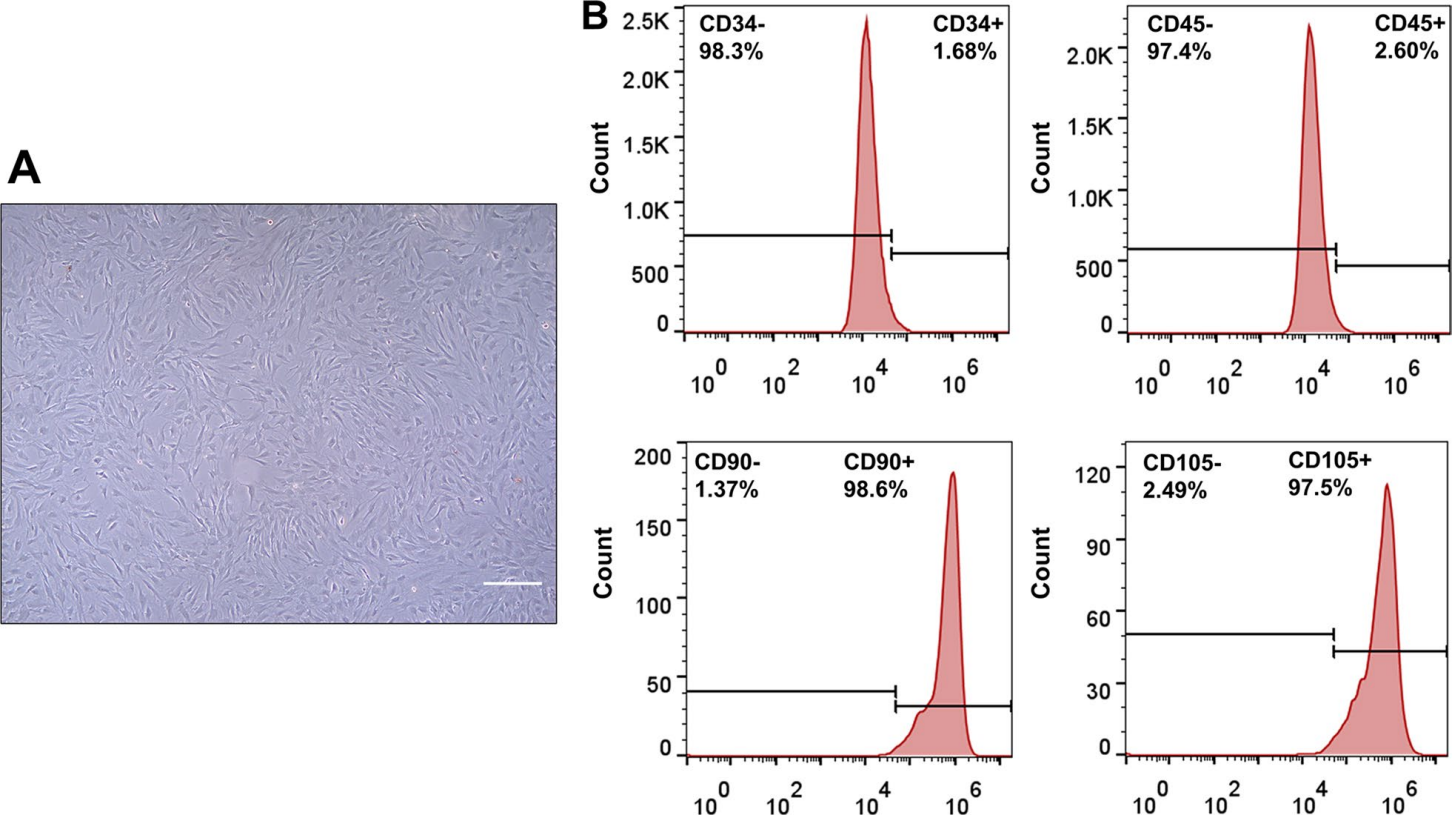
BMSC identification. **A** BMSC morphology (P3; scale bar = 200 μm). **B** Characterization of cell surface markers (CD34, CD45, CD90, and CD105) in BMSCs

Rat BMSCs had a typical spindle- and fibroblast-like shape (Fig. 1A). Immunophenotype analysis indicated that BMSCs positively expressed MSC markers CD90 and CD105 but negatively expressed hematopoietic markers CD34 and CD45, consistent with BSMC characteristics (Fig. 1B).

### BMSC osteogenic differentiation is associated with time-dependent autophagy modulation

**Fig. 2:**
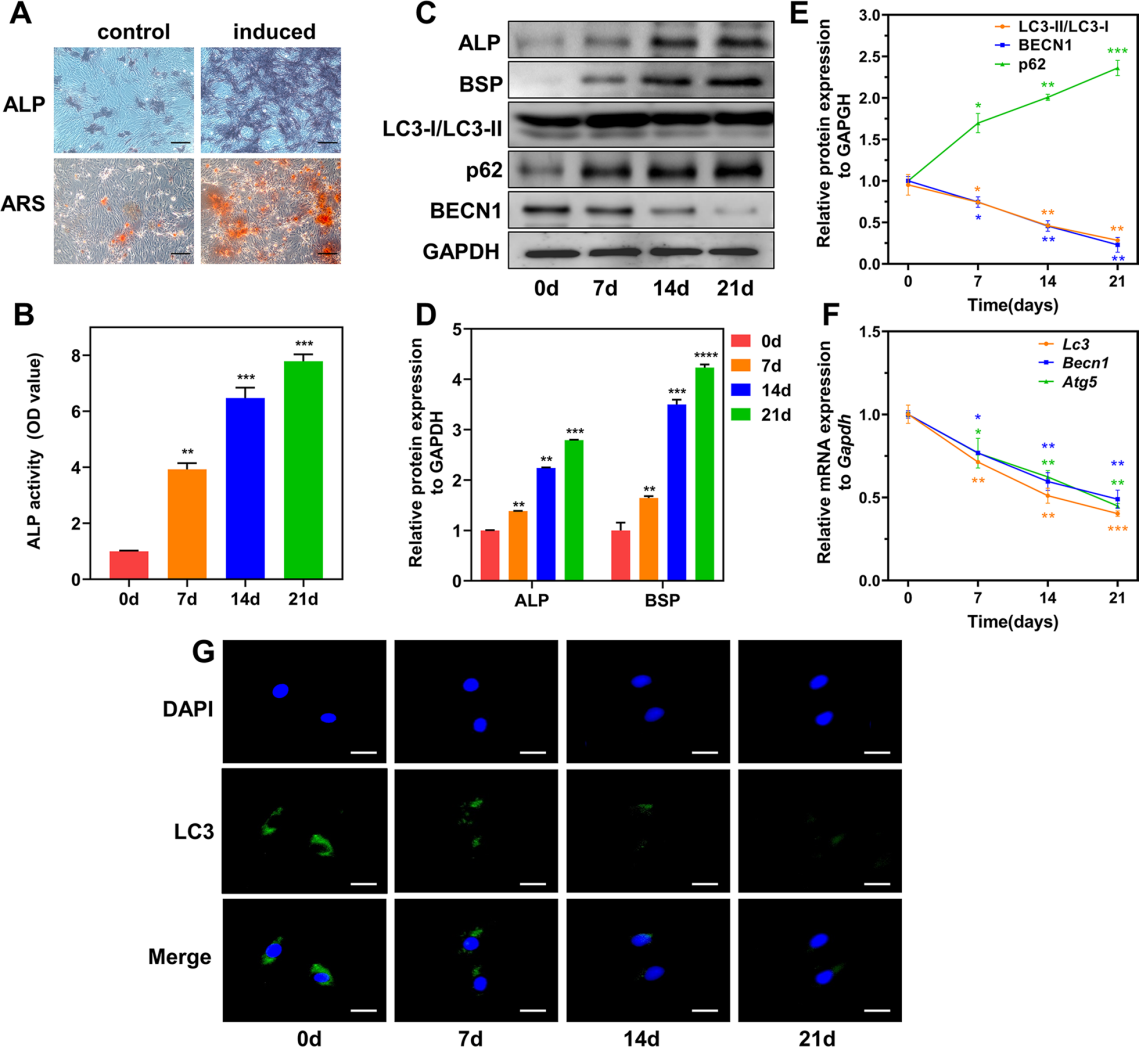
Autophagy modulation during BMSC osteogenic differentiation. **A** ALP and ARS staining in control and induced groups (scale bar = 100 μm). **B** ALP activity changes during osteogenic differentiation. **C–E** ALP, BSP, LC3-I/LC3-II, BECN1, and p62 protein levels and their quantitative analysis during osteogenic differentiation. **F** Relative *Lc3*, *Becn1*, and *Atg5* gene expression in BMSCs on d 0, 7, 14, and 21 after osteogenic differentiation. **G** LC3 immunofluorescence showing autophagosome formation on d 0, 7, 14, and 21 after osteogenic differentiation in BMSCs. Scale bar = 25 μm. *p < 0.05, **p < 0.01, ***p < 0.001, ****p < 0.0001 (vs. d 0)

Both ALP and ARS staining suggested that the induced group had greater ALP activity and calcium deposition than the control group (Fig. 2A). Moreover, increasing ALP activity was observed with increasing induction time (Fig. 2B). In addition, on days 0, 7, 14, and 21 after osteogenic induction, the expression of the osteogenesis-related proteins ALP and BSP was notably increased, indicating successful BMSC osteoblast differentiation (Fig. 2C and D).

To determine whether autophagy was involved in BMSC osteogenic differentiation, different methods were used to measure their autophagy levels during osteogenic differentiation. Changes in several critical autophagy-related proteins and genes were observed. The LC3-II/LC3-I ratio was highest on day 0, gradually decreasing in the later three differentiation stages. Autophagy cargo protein p62 levels increased in a time-dependent manner, suggesting a decline in autophagic flux. Similarly, we detected decreased BECN1 levels (Fig. 2C and E). Consistently, *Lc3*, *Becn1*, and *Atg5* expression was downregulated over time during osteogenic differentiation (Fig. 2F). Moreover, the LC3 immunofluorescence results confirmed the autophagy changes in BMSCs (Fig. 2G). Altogether, these results indicated a complex, time-dependent modulation of autophagy during BMSC osteogenic differentiation.

### Cell viability with RAPA

**Fig. 3:**
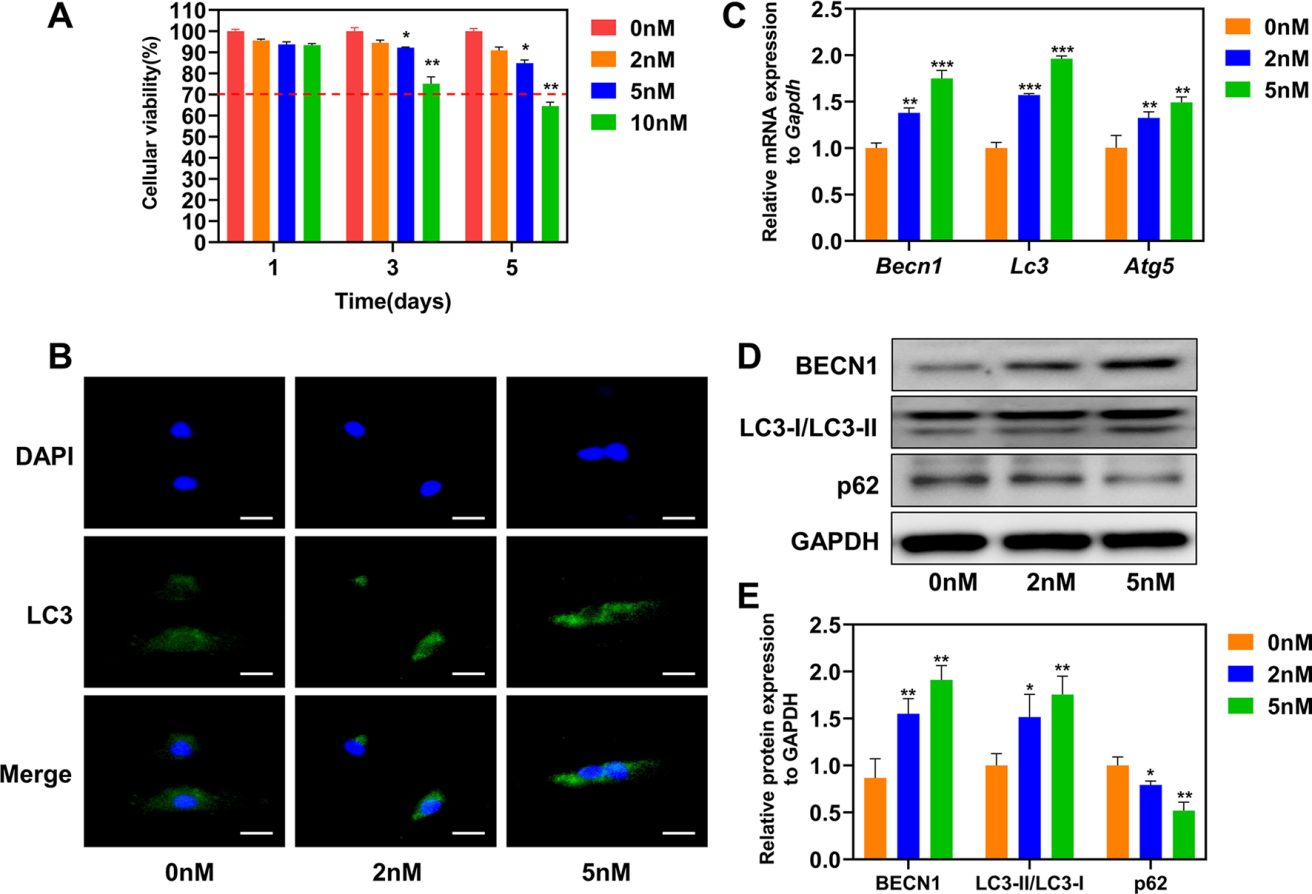
Cytotoxicity against BMSCs and autophagy levels in BMSCs after treatment with three RAPA concentrations for 24 h. **A** Cell proliferation after 1, 3, and 5 d of culturing. **B** LC3 immunofluorescence showing autophagosome formation. Scale bar = 25 μm. **C** The mRNA expression levels of autophagic markers *Becn1*, *Lc3*, and *Atg5*. **D** Protein levels of BECN1, p62, and LC3-I/LC3-II in BMSCs. **E** The LC3-II/LC3-I ratio and quantitative protein levels of BECN1 and p62. *p < 0.05, **p < 0.01, ***p < 0.001 (vs. 0 nM)

The cytotoxicity of different RAPA concentrations was evaluated using a CCK-8 assay (Fig. 3A). From day 5, 10 nM RAPA inhibited cell proliferation compared to the control group. Cell viability < 70% represents cytotoxic potential, according to the ISO standard [20]. In contrast, the cells’ survival rates in the 0 nM, 2 nM, and 5 nM groups were above 70% during the preceding five days. These results suggest that the three RAPA concentrations had no significant cytotoxicity in BMSCs. Therefore, 2 nM and 5 nM RAPA were used in subsequent experiments.

### Validation of BMSC autophagy

Different RAPA concentrations (0 nM, 2 nM, 5 nM) were used to assess autophagy in BMSCs. The autophagosomes were observed by immunofluorescence (Fig. 3B). The number of LC3 puncta in the BMSCs gradually increased as the RAPA concentration increased. Next, we investigated the effect of autophagy activation by RAPA on the protein and mRNA expression of autophagy-related markers (Fig. 3C). The expression of *Becn1*, *Lc3*, and *Atg5* increased in a dose-dependent manner. As expected, the LC3-II/LC3-I ratio and protein expression of BECN1 and p62 showed the same trend (Fig. 3D and E), indicating that autophagy increased with increasing RAPA concentration.

### Autophagy levels in BMSCs with different RAPA concentrations

**Fig. 4:**
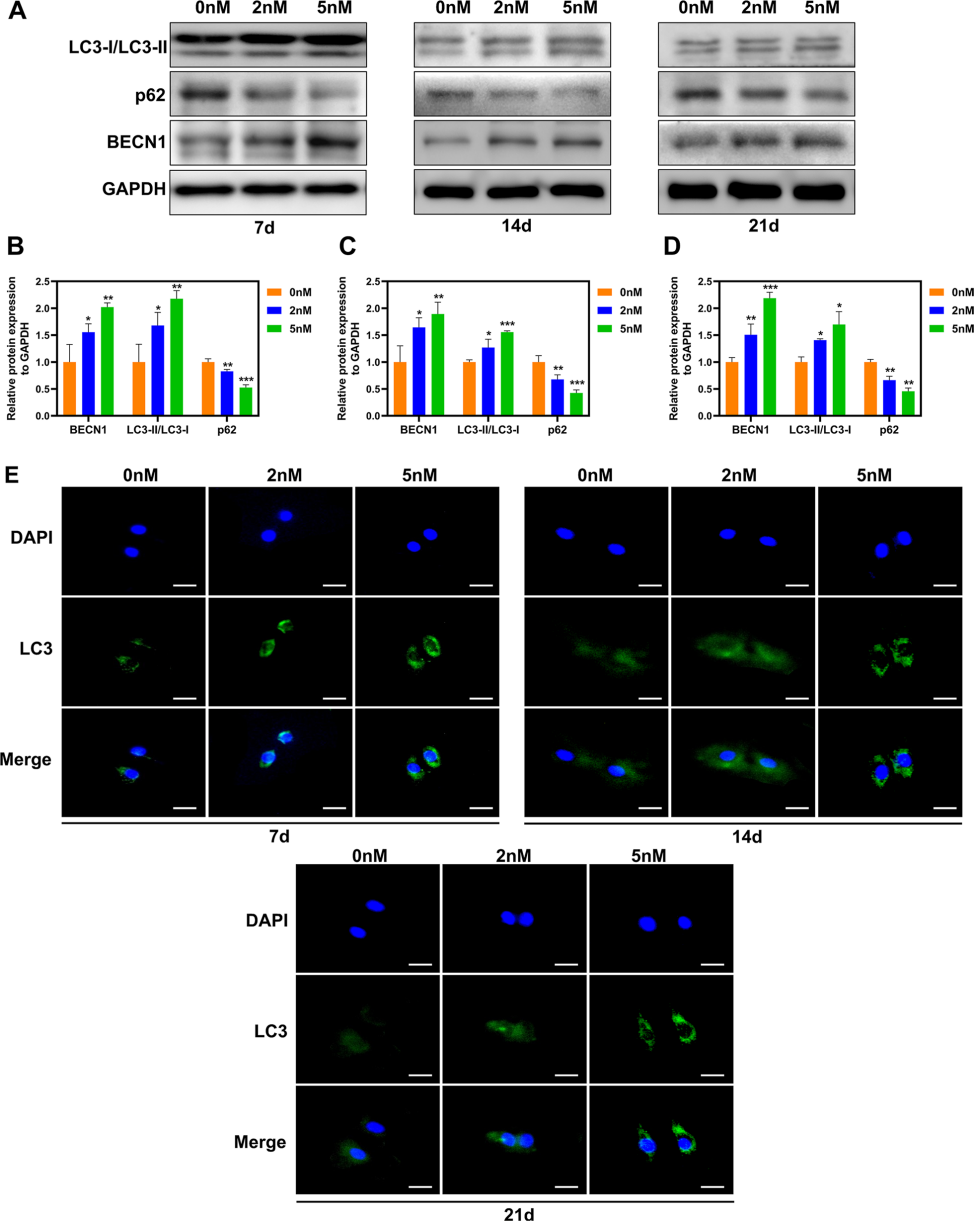
RAPA gradually induced autophagy in BMSCs during osteogenic differentiation. **A** Protein levels of BECN1, p62, and LC3-I/LC3-II on d 7, 14, and 21 after osteogenic differentiation. Quantitative BECN1and p62 protein levels and LC3-II/LC3-I ratio on d 7 (**B**), 14 (**C**), and 21 (**D**). **E** Representative LC3 immunofluorescence images for the three groups at three time points. Scale bar = 25 μm. *p < 0.05, **p < 0.01, ***, *p* < 0.001 (vs. 0 nM)

Next, to examine whether RAPA could continuously induce autophagy in BMSCs during osteogenic differentiation, the changes in several autophagy-related proteins on the 7th, 14th, and 21st days of osteogenic differentiation were detected, respectively. Compared to the control group, RAPA treatment increased the LC3-II/LC3-I ratio and BECN1 levels but reduced p62 levels in a dose-dependent manner on days 7, 14, and 21 (Fig. 4A–D). As expected, immunofluorescence results showed a significant and gradual increase in LC3 formation with increasing RAPA concentration (Fig. 4E).

### Osteogenic differentiation in BMSCs with different RAPA concentrations

**Fig. 5:**
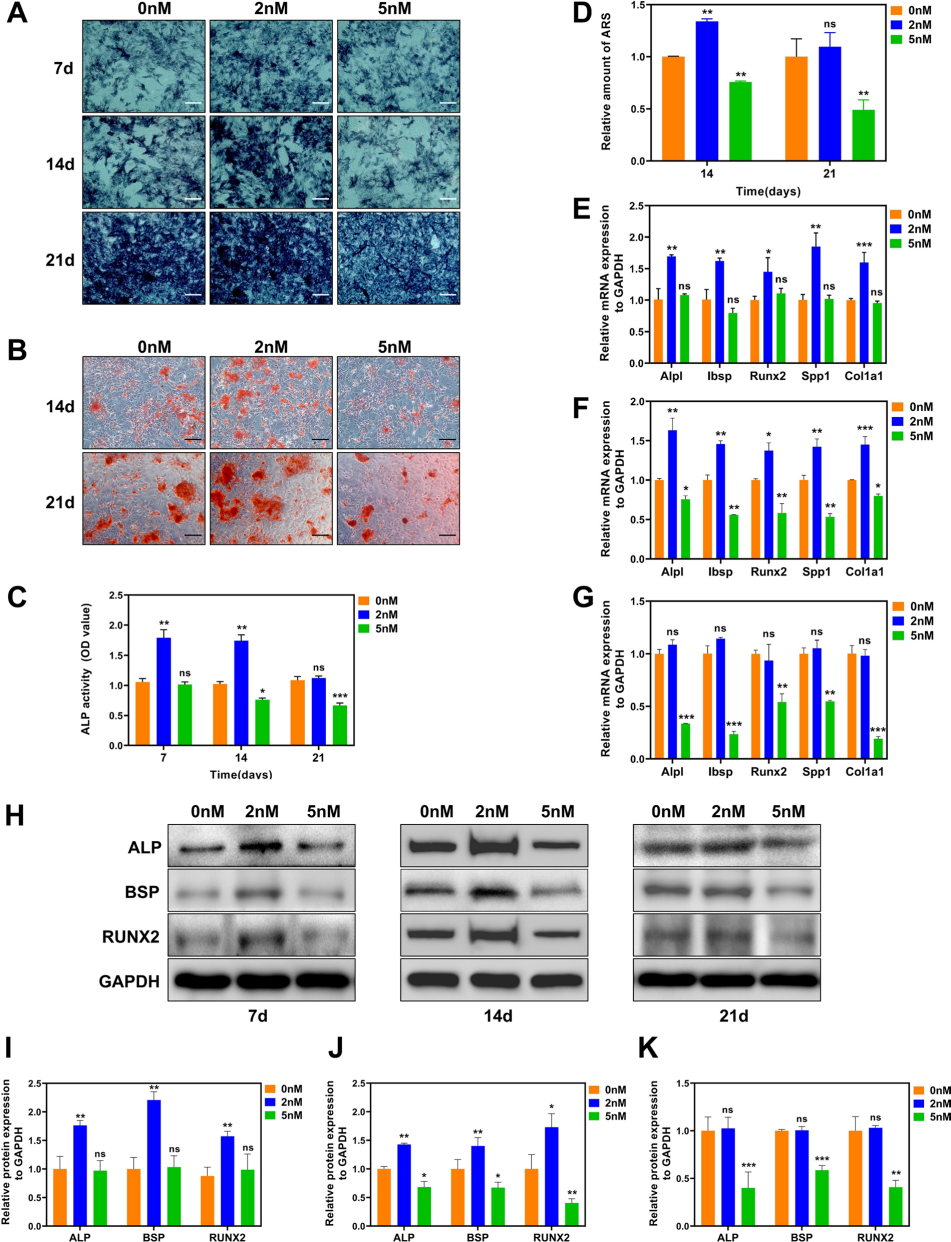
Dual osteogenesis effect of RAPA on BMSCs. **A** Representative ALP staining images for the three groups at three time points. Scale bar = 200 μm. **B** Representative images of ARS staining of BMSCs in three groups. Scale bar = 100 μm. **C** ALP activity. **D** Quantitative analysis of ARS staining of BMSCs. Gene expression levels of osteogenic markers *Alpl*, *Ibsp*, *Runx2*, *Spp1*, and *Col1a1* on d 7 (**E**), 14 (**F**), and 21 (**G**) after osteogenic differentiation.** H** Protein levels of ALP, BSP, and RUNX2 on d 7, 14, and 21 after osteogenic differentiation. Quantitative protein expression levels of ALP, BSP, and RUNX2 on d 7 (**I**), 14 (**J**), and 21 (**K**). *ns*: not significant, *p < 0.05, **p < 0.01, ***p < 0.001 (vs. 0 nM)

The effect of osteogenic differentiation was evaluated after incubating cells in an osteogenic-inducing medium containing different RAPA concentrations for 7, 14, and 21 days. Compared to the 0 nM group, ALP activity and the number of mineralization nodules increased at 7 and 14 days in the 2 nM group, while differences between the two groups were not significant at day 21 (Fig. 5A–D). Consistently, gene and protein expression levels of the osteogenic markers ALP, IBSP, RUNX2, SPP1, and COL1A1 showed the same trend between groups (Fig. 5E–K). Unlike the 2 nM group, BMSCs treated with 5 nM RAPA showed no significant difference on day 7 of osteogenic differentiation compared to the 0 nM group. However, it inhibited osteogenesis based on decreased ALP activity and ARS staining and quantification on days 14 and 21 (Fig. 5A–D). The non-significant change on day 7 and osteogenic differentiation inhibition on days 14 and 21 were further confirmed by RT-qPCR and western blots of 5 nM-treated cells (Fig. 5E–K). These findings confirm the effects of different RAPA concentrations on BMSC osteogenic differentiation, which may be related to RAPA’s promotion of different autophagic levels during osteogenic differentiation.

### RAPA promotes BMSC osteogenic differentiation through its effect on autophagy

**Fig. 6:**
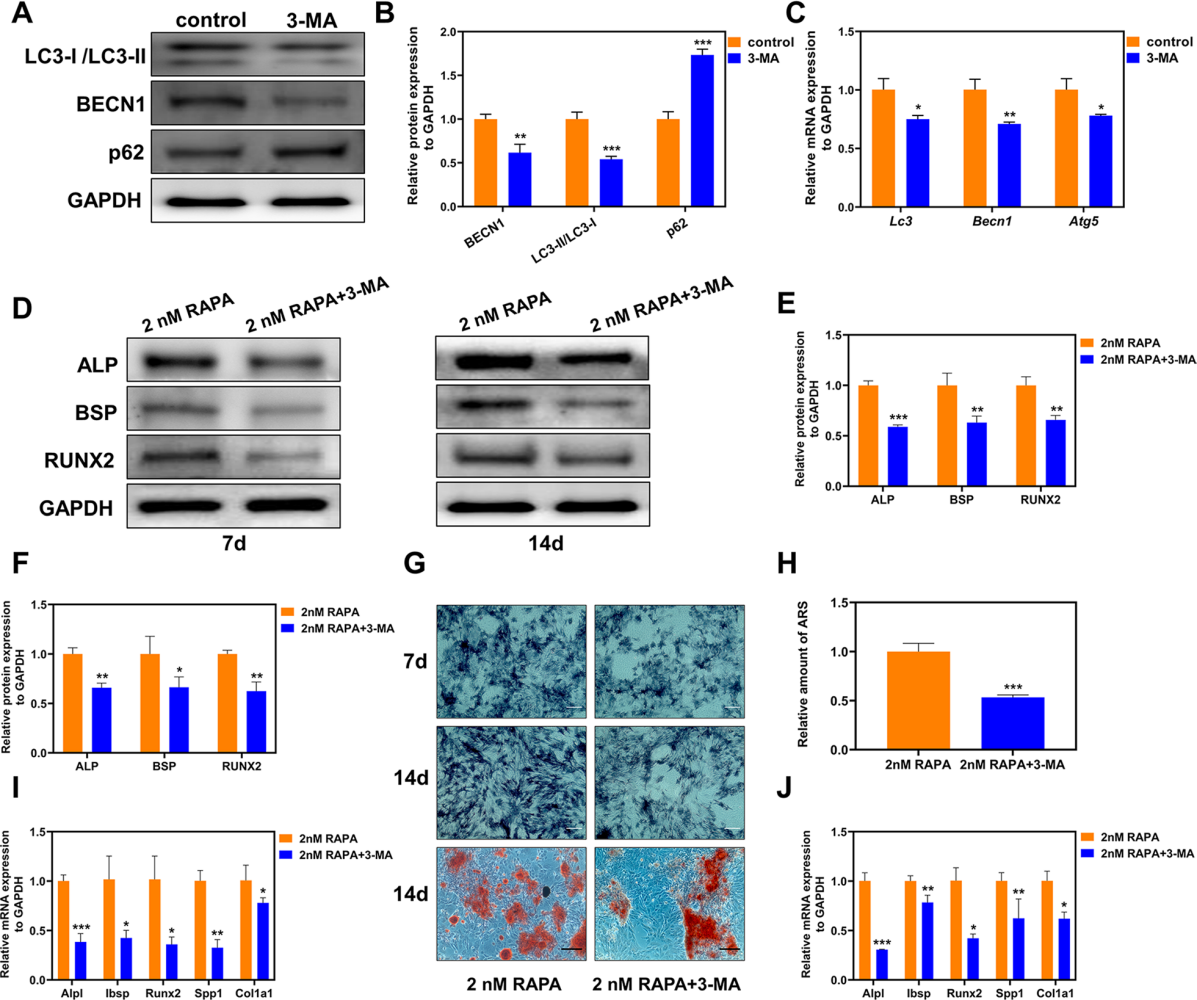
Autophagy inhibition by 3-MA prevented 2 nM RAPA-induced autophagy enhanced osteogenesis. **A** Western blot of LC3, BECN1, and p62 in BMSCs with or without 3-MA treatment for 24 h. **B** Quantitative BECN1and p62 protein levels and the LC3-II/LC3-I ratio. **C** mRNA expression levels of *Becn1*, *Lc3*, and *Atg5*. **D** Protein expression of ALP, BSP, and RUNX2 on days 7 and 14 after osteogenic differentiation. Quantitative protein expression of ALP, BSP, and RUNX2 on days 7 (**E**) and 14 (**F**). **G** Representative ALP staining images of two groups at two time points (scale bar = 200 μm) and ARS staining images on day 14 (scale bar = 100 μm). **H** Quantitative analysis of ARS staining of BMSCs. The mRNA expression levels of *Alpl*, *Ibsp*, *Runx2*, *Spp1*, and *Col1a1* on days 7 (**I**) and 14 (**J**) after osteogenic differentiation. *p < 0.05, **p < 0.01, ***p < 0.001 (vs. control or 2 nM RAPA)

Given RAPA’s apparent activation of autophagy in BMSCs during osteogenic differentiation and promotion of osteogenic differentiation, we explored whether RAPA promoted osteogenic differentiation by stimulating BMSC autophagy using the autophagy inhibitor 3-MA [6]. We found that the BECN1 protein level and LC3-II/LC3-I ratio in the 3-MA group were significantly lower than in the control group, while p62 expression was higher (Fig. 6A and B). Gene expression of *Lc3*, *Becn1*, and *Atg5* were decreased in the 3-MA group, demonstrating 3-MA suppressed autophagy (Fig. 6C). In addition, 3-MA inhibited 2 nM RAPA-enhanced osteogenesis differentiation by suppressing the protein expression of ALP, BSP, and RUNX2 on days 7 and 14 (Fig. 6D–F). ALP and ARS assays consistently indicated decreased ALP activity and mineralizing nodules in the 2 nM RAPA with 3-MA group (Fig. 6G and H). Moreover, 3-MA significantly impaired 2 nM RAPA-mediated enhancement of osteogenesis at the gene expression level (Fig. 6I and J). Altogether, these findings suggested that autophagy inhibition by 3-MA prevented 2 nM RAPA-enhanced BMSC osteogenic differentiation.

## Discussion

Autophagy is a macromolecular degradation process that enables the recycling of cytosolic proteins or dysfunctional organelles and plays a vital regulatory role in MSC differentiation [21]. Recently, several studies reported that autophagy regulates BMSC osteogenic differentiation [22, 23]. RAPA, a small molecular inhibitor, induces autophagy and has specific osteomodulatory effects. In this study, a high basal autophagy level was observed in undifferentiated BMSCs, decreasing in a time-dependent manner as osteogenic differentiation progressed. In addition, 2 nM RAPA promoted BMSC osteogenic differentiation by activating autophagy, but 5 nM RAPA inhibited this process, providing an alternative treatment approach for bone defects in orthopedics with the use of appropriate RAPA doses.

Autophagy is finely regulated to meet metabolic demands related to functional changes during cellular differentiation [24, 25]. The AMP-activated protein kinase (AMPK)-Unc51-like autophagy activating kinase 1 (ULK1) pathway positively regulates autophagy-dependent mitochondrial homeostasis in embryonic stem cells, contributing to stemness properties [26]. It has been reported that the early inhibition and late activation of autophagy play an essential role in regulating the osteogenic differentiation of dental pulp-derived MSCs [13]. Autophagy is activated when LC3-I is lipidated (LC3-II) and inserted into the autophagosome membrane [27]. Therefore, its activity is determined by the LC3-II/LC3-I ratio, with a higher ratio reflecting greater autophagy activity. p62 is an autophagic lysosomal degradation biomarker whose expression declines with the activation of autophagic flux [28, 29]. BECN1 is a crucial protein in autophagosome formation and an integral autophagy component [30]. In our work, basal autophagy decreased over time in BMSCs during osteogenic differentiation, indicating a pivotal role for autophagy in cellular differentiation. These findings are consistent with similar research on changes in autophagy in the early stage of osteogenic differentiation in dental pulp-derived MSCs [13] and the late differentiation of human BMSCs [15], suggesting that autophagy was might finely regulated to meet metabolic demands related to functional morphological changes [25]. Moreover, while our data showed alterations in autophagy during more BMSC differentiation stages, such as undifferentiated, early, mid, and late stages, and further refined autophagy’s regulatory role in stem cell differentiation, the underlying mechanisms involved in this complex, time-dependent modulation of autophagy remained to be fully explored.

Given that the basal autophagy level reduced with osteogenic differentiation, we explored the effect of the classical autophagy agonist RAPA on autophagy activation and osteogenesis during the early, mid, and late differentiation stages. RAPA, an autophagy agonist, and 3-MA, an autophagy inhibitor [6], are wide-used drug induction methods. Our results showed that RAPA could effectively induce BMSCs to upregulate their autophagy levels and stably maintain it over extended osteogenic culture times, consistent with previous studies [31]. Interestingly, RAPA has a dual role in osteogenesis. While it promotes osteogenesis in human embryonic stem cells [18] and rat bone sarcoma cells [32], its addition to fetal cranial [33] and MC3T3-E1 [34] cell cultures had osteo-inhibitory outcomes. However, the reasons behind these contrasting effects remain unclear. In our research, we further evaluated the osteogenic differentiation of BMSCs cultured with different RAPA concentrations based on alkaline phosphatase activity, the formation of mineralization nodules, and the quantification of the osteogenesis-related markers ALP, IBSP, SPP1, RUNX2, and COL1A1 at various differentiation stages. *Alpl*, *Runx2*, and *Col1a1* are expressed during the early bone formation stages, while *Spp1* and *Ibsp* are expressed in the mid-to-late stages of osteogenic differentiation, which is indispensable for differentiated osteoblast formation [35, 36]. In this study, 2 nM RAPA promoted BMSC osteogenesis in the early (day 7) and middle (day 14) osteogenic differentiation stages. However, 5 nM RAPA inhibited osteogenic differentiation, characterized by decreased alkaline phosphatase activity, formation of mineralization nodules and the gene expression of *Alpl*, *Ibsp*, *Spp1*, *Runx2*, and *Col1a1*. Our results are consistent with previous studies [18, 32–34] that showed RAPA has both stimulatory and inhibitory effects on osteogenic differentiation. The conflicting data on RAPA’s osteogenic properties could result from differences in cell type, culture media, and concentration used in the different studies.

We verified whether RAPA promoted osteogenesis by activating autophagy by using the autophagic inhibitor 3-MA to inhibit autophagy and observing BMSC osteogenic differentiation indicators. We found the osteogenesis-related genes and proteins expression levels were decreased in the 2 nM RAPA with 3-MA group. These experiments showed that 3-MA impaired RAPA’s osteogenic promotion, indicating that RAPA promotes osteogenic differentiation by promoting autophagy. Autophagy occurs at a certain level in all cells to maintain intracellular homeostasis and perform quality control of proteins and organelles [27]. Metabolic requirements associated with morpho-functional changes during cellular differentiation require fine-tuning of the autophagic pathway [24]. Therefore, 2 nM RAPA likely promoted osteogenesis by moderately and stably promoting autophagy to better meet the BMSCs’ energy requirements during osteogenic differentiation. However, paradoxically, overactivation of autophagy can lead to cellular damage [37]. Previous studies on other cells showed that excessive autophagy was a significant cause of cell death after traumatic brain injury [38]. Osteogenesis inhibition by 5 nM RAPA in this study might be caused by long-term excessive autophagy stimulation disturbing intracellular homeostasis, potentially providing a rational explanation for its dual influences on osteogenic differentiation both in previous and this study. Given the results of this study, it is more likely that RAPA’s dual effect on osteogenesis could be caused by different autophagy levels induced by different RAPA concentrations. Therefore, RAPA’s impact on osteogenesis via autophagy and the underlying mechanisms remain a promising research area.

In this study, we examined autophagy’s roles at multiple osteogenic differentiation stages, the effect of pharmacological autophagy agonist RAPA on autophagy, and its correlation with BMSC osteogenesis. However, our study has some limitations. First, the mechanisms underlying autophagy’s regulatory role in BMSC osteogenic differentiation were not investigated. Second, we did not perform in vivo studies to determine whether RAPA affects osteogenesis through autophagy, which will be the next phase of our research. Third, RAPA is a commonly used immunosuppressive drug in clinical practice [39] that has been used in immunotherapy for MSCs and bone transplantation [40, 41]. While this was only an explorative study on RAPA’s influences on osteogenesis and autophagy, our findings provide valuable insights into RAPA’s differential effects as an immunosuppressant and possible osteo-modulator in bone transplantation and regenerative medicine. As such, given this study’s results, RAPA may become a central factor for autophagy regulation, immunotherapy, and osteogenic modulation, representing a new research direction in the future. Moreover, further researches are needed to investigate the underlying autophagy mechanism in osteogenic differentiation and confirm the osteogenic promotion of RAPA via autophagy.

## Conclusions

In summary, this research demonstrated the basal autophagy of BMSCs is time-dependent decreasing during osteogenic differentiation. In addition, our data indicated that appropriate RAPA concentrations might promote BMSC osteogenic differentiation via autophagy activation.

## Abbreviations

BMSCs: Bone marrow mesenchymal stem cells
RAPA: Rapamycin
3-MA: 3-Methyladenine
MSC: Mesenchymal stem cell
mTOR: Mammalian target of RAPA
ALP: Alkaline phosphatase
ARS: Alizarin red staining
AMPK: AMP-activated protein kinase
ULK1: Unc51-like autophagy activating kinase 1
